# Effects of Lightning Corrected by the Cold Affusion

**Published:** 1833

**Authors:** 


Art. III. *Effects of Lightning corrected by the cold affusion.*—Dr. Young, of Pennsylvania, has published in the Journal to which we have just referred, a case of recovery from a stroke of lightning, by the affusion of cold water. This remedy is not new, but it may be well, occasionally, to recall the attention of the profession to its effects; as the infrequency of such accidents, manifestly contributes to our forgetfulness of the most appropriate treatment.

"An hour after the stroke, the patient was entirely insensible to every external impression, with deep, slow, interrupted respiration; the relaxation of the muscular system was complete: the pulse, as to fullness, was nearly natural, it was soft, very easily compressible, and very slow, being only about 60, as near as could be ascertained by a second watch; the pupil was sensible, though fully dilated. In less than half a minute, the patient began to toss about and moan for the first time since the accident; by a continuance of the dashing for about five minutes, she appeared so much aroused, as was evinced by crying, endeavoring to talk, though without being able, tossing, and shrinking from the cold, that we discontinued its use, had her wet clothes taken off, and had her put to bed and covered up warm. After this, she was alternately still, and inclined to doze and sleep for a minute or two at a time, then to roll from side to side, moan, and seemed in great distress; the surface was cold, but the pulse harder, more frequent and quick; the pupils were contracted, and every thing indicated a change, approaching to reaction, to have taken place; I remained with her till 2 o'clock A. M., when it was established, she could talk and explain her sensations; she complained of very severe pain in the head, and I now apprehended danger from the opposite quarter, to anticipate which, I bled her to twenty ounces by estimate, keeping my finger on the pulse, and not stopping till there was a perceptible change in it; her head was bathed with cool vinegar, and as her feet were yet cold, they were enveloped in wetted horse-radish leaves; in half an hour she sunk into a composed, comfortable sleep, from which she did not rouse till half-past 7 o'clock. Finding the pulse again too active, and the patient still complaining of some headache, I again bled her twelve ounces, ordered a dose of Epsom salts to be given in three or four hours, with some other directions concerning diet, stillness, &c. and left her, with a request to be sent for, if it was thought necessary. I heard no more from her for a week, when she had almost entirely recovered from all the effects of the injury.

Might not this cold dashing be serviceable in cases of severe concussion from falls, blows, &c. when the patient lies for hours, and even days before reaction comes on? I have not had an opportunity of trying it, since I witnessed its effects in the above case, but I think it worth a trial."

